# Global asthma prevalence in adults: findings from the cross-sectional world health survey

**DOI:** 10.1186/1471-2458-12-204

**Published:** 2012-03-19

**Authors:** Teresa To, Sanja Stanojevic, Ginette Moores, Andrea S Gershon, Eric D Bateman, Alvaro A Cruz, Louis-Philippe Boulet

**Affiliations:** 1 Child Health Evaluative Sciences, The Hospital for Sick Children, Toronto, Ontario, Canada; 2 University of Toronto, Toronto, Ontario, Canada; 3 The Institute for Clinical Evaluative Sciences, Toronto, Ontario, Canada; 4 McMaster University, Hamilton, Ontario, Canada; 5 Sunnybrook Health Sciences Centre, Toronto, Ontario, Canada; 6 University of Cape Town, Cape Town, South Africa; 7 ProAR - Núcleo de ExcelênciaemAsma, Faculdade de Medicina da Bahia, UFBA, Salvador, Brazil; 8 Laval University, Quebec City, Quebec, Canada; 9 Child Health Evaluative Sciences, The Hospital for Sick Children, 555 University Avenue, Toronto, Ontario M5G 1X8, Canada

## Abstract

**Background:**

Asthma is a major cause of disability, health resource utilization and poor quality of life world-wide. We set out to generate estimates of the global burden of asthma in adults, which may inform the development of strategies to address this common disease.

**Methods:**

The World Health Survey (WHS) was developed and implemented by the World Health Organization in 2002-2003. A total of 178,215 individuals from 70 countries aged 18 to 45 years responded to questions related to asthma and related symptoms. The prevalence of asthma was based on responses to questions relating to self-reported doctor diagnosed asthma, clinical/treated asthma, and wheezing in the last 12 months.

**Results:**

The global prevalence rates of doctor diagnosed asthma, clinical/treated asthma and wheezing in adults were 4.3%, 4.5%, and 8.6% respectively, and varied by as much as 21-fold amongst the 70 countries. Australia reported the highest rate of doctor diagnosed, clinical/treated asthma, and wheezing (21.0%, 21.5%, and 27.4%). Amongst those with clinical/treated asthma, almost 24% were current smokers, half reported wheezing, and 20% had never been treated for asthma.

**Conclusions:**

This study provides a global estimate of the burden of asthma in adults, and suggests that asthma continues to be a major public health concern worldwide. The high prevalence of smoking remains a major barrier to combating the global burden of asthma. While the highest prevalence rates were observed in resource-rich countries, resource-poor nations were also significantly affected, posing a barrier to development as it stretches further the demands of non-communicable diseases.

## Background

Asthma is a major cause of disability, health resource utilization and poor quality of life for those who are affected. It is the most common chronic disease among children and young adults, particularly because of its early onset (one out of four individuals in the general population develops asthma before the age of 40 years) [1], it accounts for considerable healthcare costs and loss of work productivity [2]. In 2004, Masoli et al., and the Global Initiative for Asthma (GINA) combined data from the Phase 1 International Study of Asthma and Allergies (ISAAC) study collected in 1992-1996 and the European Community Respiratory Health Survey (ECRHS) in 1988-1994 to generate global estimates of asthma burden, which suggested that asthma prevalence ranged from a low of 0.7% in Macau to 18.4% in Scotland [3-6]. This report estimated that 300 million people worldwide had asthma, and projected that this number would increase to 400 million by 2025, as countries became more urbanized [4].

The GINA report however was at best a general estimate, as the data on which it was based came from different surveys collected between (1988 and 1996) using different sampling methodologies and asthma definitions, and recruited different age groups, The World Health Survey (WHS) [7], designed and implemented by the World Health Organization (WHO) from 2002 to 2003, employed a standardized methodology to collect information from which to estimate health of populations which would allow within and between country comparisons. This data was intended to inform policy in a wide range of countries from six continents around the world. The WHS is public data available through the WHO upon request and has previously been used to estimate the burden of other chronic diseases and risk factors for these diseases [8-13]. This standardized cross-sectional survey was implemented by 70 of the 192 WHO member states, and constituted the largest multi-country survey of asthma in adults to date. We used WHS data to estimate and compare the global and country-specific burden of asthma.

## Methods

### World health survey (WHS)

A stratified probability sampling design, where the sampling frame covered 100% of the country's eligible adults ≥ 18 years of age was used in each of the countries. The sample was stratified by sex, age and urban/rural living strata [7]. A multistage cluster design was used in all countries except Australia, China, Comoros, Congo, Cote d'Ivoire, Croatia, India, and Russia, where post-stratification probability weight were not available (simple weights were used), and Austria, Belgium, Denmark, Germany, Guatemala, Netherlands, Slovenia, UK, and Zambia where no probability weights were available.

Each respective Ministry of Health of the 70 member states who elected to participate in the WHS was responsible for designing the local sampling strategy and administering the standardized questionnaires. The sample size for each participating country ranged from 1,000 to 10,000 participants; each country chose a sample size based on their needs, amount of detail required and feasibility/survey costs. Each of the survey modules were pilot tested in 12 countries. Translated surveys were administered by trained personnel either face-to-face or by telephone (Australia, Israel, Luxembourg, and Norway), using both paper and electronic questionnaire formats, depending on feasibility [7]. Training courses for participating countries were run by WHO regional offices, and quality standards set by the WHS Quality Assurance Standards & Guidelines committee were monitored by external peer review. A detailed country report, providing details about the survey, is freely available on the WHS/WHO website.

### Definitions of asthma

A strict definition of asthma based solely on doctor diagnosis may be useful in some clinical settings in developed countries; however, in developing countries it may vary greatly depending on the context, availability, and access to health care and medications. A combination of diagnosis and/or treatment for asthma may more accurately classify individuals with active asthma. Since diagnosis and the availability of treatment may be challenging in resource-poor countries, a broader definition which includes respiratory symptoms, in addition to diagnosis and treatment received may yield a higher sensitivity in identifying individuals with asthma. Therefore, in this study, we estimated and compared the global burden of asthma using three definitions of asthma. The first definition was *doctor diagnosed asthma *which is based on the question "*Have you ever been diagnosed with asthma*?" The second definition was *clinical asthma *which was based on doctor diagnosed asthma and/or a positive response in either of two questions "*Have you ever been treated for asthma*" or "*Have you been taking any medications or treatment for asthma during the last 2 weeks?*" The third definition, *symptoms of asthma*, was based on doctor diagnosed asthma, clinical asthma and/or a positive response to "*During the last 12 months have you experienced attacks of wheezing or whistling breath*?" The WHS survey questions were similar to those used by the ISAAC and ECRHS surveys [3,5,6]. To avoid confusion between asthma and Chronic Obstructive Pulmonary Disease (a disease most prevalent amongst older adults); we limited our study population to individuals aged 18 to 45 years.

### Definition of smoking

Current smoking was defined based on a positive response to the question "*Do you currently smoke any tobacco products such as cigarettes, cigars, or pipes*?".

### Regional differences

Estimates of asthma prevalence were calculated for each participating country as well as for each WHO region. Six regions using the WHO definitions were included: Africa (includes 18 countries), Americas (7), Eastern Mediterranean (4), Europe (30), South East Asia (5), and Western Pacific (6) [7,14].

### Statistical analyses

All analyses were conducted using STATA statistical software [15]. Country-specific prevalence estimates were obtained by applying survey weights for complex sampling designs. No weights were applied for pooled regional analyses [11,13]. Given the different age-distributions in each of the participating countries, we age-standardized the country specific prevalence estimates. However, since the standardized estimates were similar to the unadjusted estimates (Mean Difference: -0.04; 95% Limits of Agreement: -0.07; 0.62), we presented the unadjusted results.

### Missing data

The risk factor module included questions related to tobacco use and exposure to pollutants and was available for 53 countries [16]. Smoking data was missing for Austria, Australia, Belgium, Denmark, Finland, France, Germany, Greece, Ireland, Israel, Italy, Luxembourg, Netherlands, Norway, Portugal, Sweden, and the United Kingdom. These countries account for only 9,803 of the 181,042 individuals (5.41%) who completed the survey.

### Ethics

The study materials and methods were approved by the research ethics board at the Hospital for Sick Children (REB # 1000025091).

## Results

Of the 181,042 individuals who completed the WHS, a total of 178,215 (98.4%) adults in 70 countries responded to the questions related to asthma diagnosis and respiratory symptoms. The median survey response rate at the household level was 93.0%, whereas the median response rate at the individual level was 99.0%. Country-specific response rates can be found on the WHS website [16]. Overall, the question-specific response rates was high, ranging from 73.3 to 97.7%. The lower response rates observed for clinical asthma was due to low response rates in the Americas (34.1%). All other regions had response rates above 70% for clinical asthma.

### Global burden of asthma

**Figure 1:**
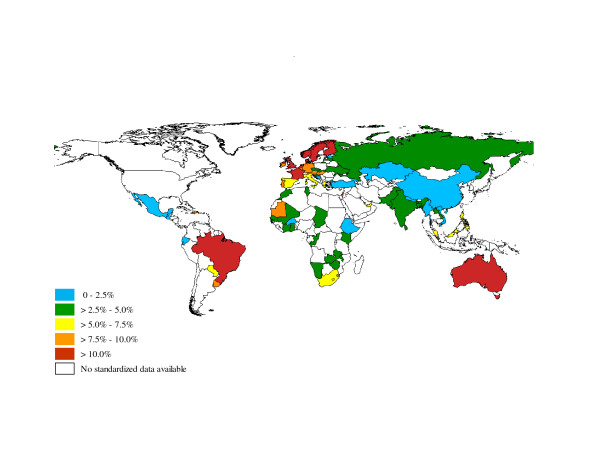
**Worldwide prevalence of clinical asthma**.

**Figure 2:**
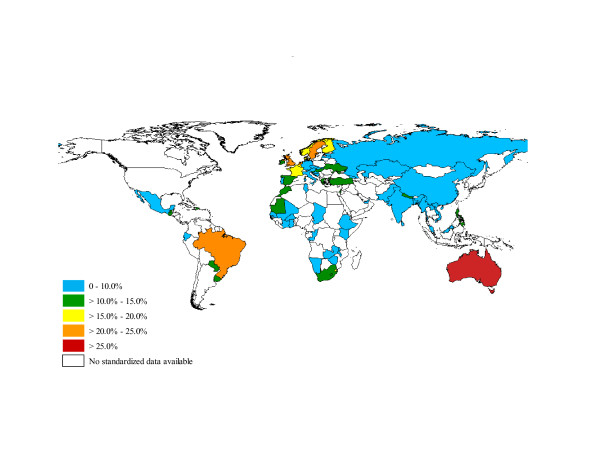
**Worldwide prevalence of wheezing asthma**.

The global prevalence of doctor diagnosed asthma in adults was estimated to be 4.3% (95% CI: 4.2; 4.4). The prevalence of doctor diagnosed asthma varied widely amongst the 70 participating countries, ranging from 0.2% in China to 21.0% in Australia (Table 1 and Figure 1). Using a less stringent definition, the global prevalence of clinical asthma (or treated asthma) was 4.5% (95% CI: 4.4; 4.6). The prevalence of clinical asthma also varied widely amongst the 70 participating countries, ranging from 1.0% in Vietnam to 21.5% in Australia, representing a 21-fold global variation (Table 1 and Figure 2). The five countries with the highest prevalence of clinical asthma were Australia (21.5%), Sweden (20.2%), UK (18.2%), Netherlands (15.3%), and Brazil (13.0%). Finally, using the least stringent definition, the global prevalence of wheezing was estimated to be 8.6% (95% CI: 8.5; 8.7). The prevalence of wheezing had a 15-fold variation across the world (Table 1 and Figure 2), with the highest rates observed in Australia (27.4%), the Netherlands (22.7%), the United Kingdom (22.6%), Brazil (22.6%), and Sweden (21.6%).

**Table 1:** Region and country-specific estimates of asthma prevalence by 3 definitions

		**Asthma Prevalence (%)**^**2**^		
**Region**^**1**^	**Country**	**Doctor** **Diagnosed Asthma**	**Clinical Asthma**	**Wheezing Symptoms**

Africa	Burkina Faso	2.02	2.26	5.32

	Chad	3.68	3.94	7.64

	Comoros^3^	7.55	7.80	12.85

	Congo^3^	4.65	4.79	7.93

	Cote d'Iviore^3^	4.22	4.59	7.70

	Ethiopia	2.00	2.00	5.53

	Ghana	3.65	3.77	4.88

	Kenya	2.86	3.12	6.22

	Malawi	4.62	4.67	7.76

	Mali	2.65	2.82	4.77

	Mauritania	6.95	7.54	11.78

	Mauritius	3.88	3.92	6.88

	Namibia	3.16	3.39	8.14

	Senegal	3.43	3.72	8.40

	South Africa^5^	5.92	6.09	12.40

	Swaziland^5^	8.74	9.69	15.37

	Zambia^4^	2.83	2.96	6.25

	Zimbabwe	2.28	2.52	5.48

	*Regional Sub-total*	*3.94*	*4.19*	*7.75*

Americas	Brazil	12.44	12.98	22.56

	Dominican	9.63	9.97	12.39

	Ecuador	2.03	2.13	3.83

	Guatemala^4^	2.26	2.42	11.95

	Mexico	2.39	2.39	3.87

	Paraguay	6.08	6.40	12.74

	Uruguay	8.60	9.10	12.02

	*Regional Sub-total*	*4.27*	*4.40*	*7.61*

Eastern Mediterranean	Morocco^5^	2.76	2.84	11.65

	Pakistan	3.12	3.13	5.02

	Tunisia	2.74	2.79	7.21

	United Arab Emirates	5.30	2.79	7.21

	*Regional Sub-total*	*2.93*	*2.99*	*7.60*

Europe	Austria^4^	7.46	7.63	9.48

	Belgium^4^	9.83	10.00	17.22

	Bosnia Herzegovina^5^	1.30	1.41	4.01

	Crotia^3^	4.38	4.57	8.66

	Czech Republic	4.56	4.71	6.32

	Denmark^4^	9.50	10.19	15.40

	Estonia	2.00	1.99	6.94

	Finland	9.39	10.24	17.19

	France	10.43	10.59	15.20

	Georgia	2.09	2.15	4.83

	Germany^4^	7.58	7.55	9.25

	Greece^4^	6.60	6.84	10.14

	Hungary	7.66	7.66	14.72

	Ireland	9.41	9.19	11.39

	Israel	7.59	8.54	14.98

	Italy^4^	6.05	6.26	8.98

	Kazakhstan	1.43	1.47	3.36

	Latvia	2.70	2.70	5.90

	Luxembourg	9.16	9.44	16.63

	Netherlands^4^	15.17	15.32	22.71

	Norway	11.05	12.32	15.05

	Portugal	7.83	7.83	8.72

	Russia^3^	2.50	2.57	4.98

	Slovakia	4.11	4.10	7.41

	Slovenia^4^	8.70	8.66	11.91

	Spain^5^	6.79	7.12	12.78

	Sweden	20.09	20.18	21.60

	Turkey	2.06	2.11	11.34

	UK^4^	17.59	18.15	22.59

	Ukraine	2.77	2.90	11.13

	*Regional Sub-total*	*5.1*	*5.28*	*10.71*

South East Asia	Bangladesh	2.91	3.23	8.63

	India^3^	3.16	3.30	9.63

	Myanmar	2.36	2.41	3.47

	Nepal	2.04	2.16	14.37

	Sri Lanka	2.60	2.75	6.35

	*Regional Sub-total*	*3.24*	*3.39*	*9.71*

Western Pacific	Australia^3^	20.96	21.51	27.39

	China^3^	0.19	1.42	1.73

	Laos	2.72	3.02	5.16

	Malaysia	5.21	5.51	7.55

	Philippines	7.21	7.46	11.01

	Vietnam	0.82	1.04	2.05

	*Regional Sub-total*	*5.85*	*6.17*	*8.88*

Worldwide (95%CI)		4.27 (4.17; 4.36)	4.46 (4.36; 4.55)	8.61 (8.48; 8.74)

^1 ^World Health Organization definition of regions

^2 ^All asthma prevalence estimates were calculated using post satisfaction weights unless otherwise indicated

^3 ^Post stratification weights were not available from these countries, so sampling weights were used in calculating the prevalence estimates

^4 ^Weights were not provided these countries

^5 ^Standard errors were missing due to a sampling unit

### Regional differences in clinical or treated asthma

#### Overall

The prevalence of clinical asthma in adults did not differ greatly between regions (Table 2). The highest prevalence of clinical asthma was in the Western Pacific region (6.2%) which was largely contributed by the high prevalence in Australia. The prevalence of clinical asthma was similar in rural (4.86%) and urban (4.91%) residents in all regions except the Western Pacific (p-value: 0.840). The largest differences between rural (3.7%) and urban (5.1%) were observed in the Americans.

**Table 2:** Prevalence of clinical asthma, current smoking, symptoms and treatment by regions

			% Among Clinical Asthma Population Reported Symptoms in Last 12 Months		
**Country Regions**	**Prevalence (%)** **of Clinical Asthma**	**% Current Smokers**	**% Current Smokers**	**Asthma Ever Treated**	**% Wheezing**

Africa	4.19	12.65	13.13	78.85	53.20

Americas	4.40	22.20	23.28	82.62	38.12

Eastern Mediterranean	2.99	19.41	17.52	85.52	54.45

Europe	5.28	39.25	35.85	90.24	49.64

South East Asia	3.39	30.23	34.11	78.00	57.86

Western Pacific	6.17	31.46	28.38	77.11	50.84

Global	4.46	23.46	23.33	81.91	49.69

##### Asthma symptoms

Almost half (49.7%) of individuals living with clinical asthma had experienced wheezing in the last 12 months, the highest prevalence reported being from South East Asia (57.9%).

##### Asthma treatment

Almost a fifth of those with clinical asthma had never received treatment for asthma in their life.

##### Prevalence of smoking

The prevalence of smoking in the population with clinical asthma (23.3%) was not different from that of the overall global prevalence of smoking (23.5%). In Europe and South East Asia, more than one third of the population with clinical asthma was currently smoking. There was no association between prevalence of smoking amongst individuals with asthma and the country level prevalence of asthma.

## Discussion

Using standardized WHS data obtained from the WHO, we estimated that the global prevalence of clinical asthma in adults was 4.5% and varied by as much as 21-fold amongst 70 participating countries. Amongst the population living with clinical asthma, almost one in four was a current smoker, one in two reported wheezing in the past 12 months, but one in five had never received asthma treatment. The WHS is the first standardized, representative survey which included population-based data regarding respiratory symptoms and treatment permitting estimation of the global burden of asthma in adults. This study provides the most current global estimates of the burden of asthma and shows that asthma continues to be a major public health problem worldwide.

We estimated the prevalence of asthma using three definitions ranging from the most stringent - self-reported doctor diagnosis, to the most inclusive definition - self-reported wheezing. Clinical history in combination with a reversible airway obstruction as measured by a pulmonary function test is the gold standard for diagnosing asthma. However, implementing such a standard to identify individuals with asthma is impractical considering the scale of the sample of this study, and would be extremely costly and time consuming. Our questionnaire-based classifications offer a reasonable, feasible and practical alternative. While the respiratory symptoms definition may overestimate the global asthma prevalence, the clinical definition likely underestimates disease burden in resource-poor countries with inadequate access to health care facilities and treatments. Our analyses focused mainly on the clinical asthma definition, which identifies asthma burden based on diagnosis and/or treatment. This definition likely yields a lower false positive rate compared to a symptom-based definition [17].

Compared with the asthma estimates previously reported (Table 3), our asthma estimates are more up-to-date, are based on consistent data contributed by a large number of developing countries, and include estimates for both rural and urban dwellers. Country and regional differences highlight the need for locally tailored interventions and initiatives to address the specific risk factors and needs. While not directly comparable due to differences in methods used, our country-specific estimates are broadly similar to those presented in the GINA report [4]. We also observed that the prevalence of asthma varied greatly between countries, with the highest prevalence observed in resource-rich countries [4,6]. The ISAAC study also employed a standardized global survey and was implemented locally, but the target population was children only. Unlike the WHS, some countries in the ISAAC study used convenience samples and were not necessarily multi-staged or stratified to be representative of the entire country. The ISAAC study used a video questionnaire to help reduce misclassification due to translation of wheezing symptoms; this, however, was not practical for the WHS. The ECRHS was limited to developed countries, and therefore cannot be used to infer global figures. Finally, since the GINA Burden of Asthma 2004 estimates were a retrospective combination of the ISAAC and ECHRS surveys, the country specific estimates are not necessarily representative of the entire population, and the averaging to different surveys from the same country introduces bias since different instruments were used.

**Table 3:** Summary of prevalence of asthma reported in the literature

Authors	Data Collection Period	Study & Survey Used	Definitions of Asthma	Sample Size	Countries Included	Age Groups	Findings of Asthma Prevalence
ECRHS (1996)^5^	1988-1994	ECRHS	An asthma attack in the last 12 months or currently taking asthma medication	137,619	12	20-44	Ranged from 2.0% in Estonia to 11.9% in Australia with a median of 4.5%

ISAAC Steering Committee (1998)^3^	1992-1996	ISAAC Phase 1	Self-reported ever had asthma	721,601	56	6-7 and 13-14	11.3% in children aged 13 14 years and 10.2% in children aged 6-7 years

ISAAC Steering Committee (1998)^6^	1992-1996	ISAAC Phase 1	Wheezing or whistling in the chest in the last 12 months	463,801	55	13-14	Ranged from 1.6% in Indonesia to 36.8% in the UK

Masoli et al. (2004)^4^	1988-1996	ISAAC and ECRHS	Various depending on the survey and country	Not reported	76	13-14 and 20-44	Ranged from 0.7% in Macau to 18.4% in Scotland

Lai et al. (2009)^18^	2000-2003	ISAAC Phase 3	Wheeze in the past 12 months	1,187,496	98	6-7 and 13-14	14.1% in children 13-14 years and 11.5% in children 6-7 years

Sembajwe et al. (2010)^13^	2002-2003	WHS	Doctor diagnosis	308,218	64	18-99	Ranged from 1.8% in Vietnam to 32.8% in Australia with an overall 6.0%

To et al. (2011) (Current study)	2002-2003	WHS	Doctor diagnosis, clinical and symptoms of asthma	177,496	70	18-45	4.3% doctor diagnosed asthma, 4.5% clinical asthma and 14.4% symptoms of asthma

In 2010, Sembajwe et al. used the WHS data, and reported variations in wheezing symptoms and doctor diagnosed asthma prevalence across world regions relating them to national income [13]. All subjects aged 18 to 99 years from 64 countries were included in their study. They reported a 6% prevalence of doctor diagnosed asthma and 9.2% for current wheezing, which does not agree with our findings. The major differences between their findings and ours may be attributed to differences in study population included (ours included all 70 participating countries but limited it to participants aged 18 to 45 years old). Since our prevalence estimates were lower, it suggests that their estimates may have been biased by the inclusion of subjects with COPD as asthma.

Using the WHS data to measure the global burden of asthma offers several strengths. Firstly, the same standardized questionnaires were applied to all individuals who participated. Secondly, the survey was administered using multi-staged random sampling in most of the sites making the country-specific estimates representative of the whole population. The survey in each country was also stratified by age, sex and rural/urban residence, further improving the generalizability of our findings. Nevertheless, the WHS data only included adults, and likely underestimates the global burden, since asthma is more prevalent in children. Canada and the United States are notable absences from the Survey, however participation was voluntary and these countries elected not to take part. Therefore, our estimates likely underestimate the total global burden of asthma, but the sample of countries included in the WHS is sufficient to make statistically sound global inferences

Our results highlight that asthma continues to be a major public health concern worldwide. Applying our 4.5% clinical asthma prevalence to the current world population of 7 billion translates to 315 million individuals with asthma. However, using our 8.6% self-reported prevalence of asthma symptoms, we estimated that nearly 623 million individuals are currently living with some level of asthma-related symptoms worldwide. While proper long-term management of asthma will allow individuals with asthma to achieve good levels of control enabling them to live with good quality of life, our data indicates that asthma control is not optimal in many countries. Worldwide, nearly half of the asthma population reported wheezing in the last 12 months, and only a moderate proportion had been diagnosed and/or received treatment. In addition, the high prevalence of smoking continues to be one of the major barriers in combating the global burden of asthma. While the highest overall prevalence of asthma was observed in resource-rich countries, many resource-poor nations also have a high prevalence of this disease. This is of concern because in most such countries, resources are consumed by the pressing demands of infectious diseases and the need to provide primary care for the broader population. In many countries there is little, if any provision of the essential medications that at both individual and population level can lead to very satisfactory control of asthma. Uncontrolled asthma poses an extra weight in the burden of non-communicable disease, which constitutes a major barrier for development.

## Conclusions

The asthma statistics from the WHS presented here may be useful to health policy and decision makers, clinicians, and researchers in designing programs and plans of action to address risk factors such as smoking and improve quality of provided for asthma and reduce the burden that it presents, through provision of treatment that is adequate, accessible, and effective.

## Competing interests

The authors declare that they have no competing interests.

## Authors' contributions

TT obtained data from the World Health Organization and is responsible for the concept and design of the study. TT and SS participated in the interpretation of data, analysis, and drafting the manuscript. GM participated in the interpretation of data and drafting the manuscript. AG, AC, EB, and LPB assisted with reviewing and revising the final manuscript. All authors approved the manuscript as submitted and take full responsibility for the manuscript.

## Pre-publication history

The pre-publication history for this paper can be accessed here:

http://www.biomedcentral.com/1471-2458/12/204/prepub
