# Correction to: Global asthma prevalence in adults: findings from the cross-sectional world health survey

**DOI:** 10.1186/s12889-021-11859-6

**Published:** 2021-10-08

**Authors:** Teresa To, Sanja Stanojevic, Ginette Moores, Andrea S. Gershon, Eric D. Bateman, Alvaro A. Cruz, Louis-Philippe Boulet

**Affiliations:** 1 Child Health Evaluative Sciences, The Hospital for Sick Children, Toronto, Ontario Canada; 2 University of Toronto, Toronto, Ontario Canada; 3 The Institute for Clinical Evaluative Sciences, Toronto, Ontario Canada; 4 McMaster University, Hamilton, Ontario Canada; 5 Sunnybrook Health Sciences Centre, Toronto, Ontario Canada; 6 University of Cape Town, Cape Town, South Africa; 7 ProAR - Núcleo de ExcelênciaemAsma, Faculdade de Medicina da Bahia, UFBA, Salvador, Brazil; 8 Laval University, Quebec City, Quebec, Canada; 9 Child Health Evaluative Sciences, The Hospital for Sick Children, 555 University Avenue, Toronto, Ontario M5G 1X8 Canada

**Correction to: BMC Public Health 12, 204 (2012)**

**https://doi.org/10.1186/1471-2458-12-204**

This article [1] has been corrected to acknowledge that Figs. 1 and 2 have been reproduced with permission of the© ERS 2021: European Respiratory Journal Feb 2010, 35 (2) 279–286; **DOI:** 10.1183/09031936.00027509
